# The American Public Health Association

**Published:** 1894-11

**Authors:** 


### The American Public Health Association.

The following resolutions, looking to the better protection of the public health, were introduced at the recent meeting of the A. P. H. A. held at Montreal, Canada. (*From Journal American Medical Association*):

Dr. Arthur R. Reynolds, Commissioner of Health, Chicago, said, in accordance with the suggestion of the reader of the paper, he desired to offer the following resolution:

"*Resolved*, That the American Public Health Association, in convention assembled, approve and recommend the enactment of sanitary plumbing and drainage laws in the future construction of our buildings, for the better protection of the health of our people."

Under the rules, this was referred to the Executive Committee without debate.

Dr. E. R. Campbell, of Bellows Falls, Vt., drew attention to the last paper read at last evening's session. He moved the following resolution:

"*Resolved*, That the American Public Health Association, in convention assembled, records its protest against the use of alcoholic liquor as a beverage, especially among the young, believing that such use is attended with great danger to the health, the individual and society."

The resolution was referred to the Executive Committee.

The following resolution, moved by Dr. Henry Sewell, of Denver, Col., was also referred to the Executive Committee:

"*Resolved*, That a committee, consisting of not more than five members, be appointed by the President of the American Public Heath Association to consider the best form of sanatoria for consumptive invalids, and the most favorable locations for the same within the United States, the report of the committee to be made at the meeting of the Association held in 1895."

Dr. George Homan, of St. Louis, Mo., offered the following resolution:

"WHEREAS, It is the sense of the American Public Health Association that the pollution of potable waters in America has reached such a point that the national government should be asked to take cognizance of the matter with the view of devising means of prevention and relief; therefore be it

"*Resolved*, That this Association memorialize the Congress of the United States and ask that they shall authorize the appointment of a competent commission clothed with power to fully investigate the whole subject of the pollution of rivers and lakes by municipal and manufacturing waste, and provided with sufficient means to enable them to conduct the examination in such manner as shall be deemed best, the result of said examination to be published, from time to time, for the public information."

Which was referred, without debate, to the Executive Committee, owing to the lateness of the hour of adjournment at last evening's session.

Dr. S. R. Olliphant, of New Orleans, President Louisiana State Board of Health, offered the following resolution which was referred to the Executive Committee:

"*Be it Resolved*, That it is the sense of this Association, that federal surveillance, control, or interference with State quarantine service, as maintained, is unwarranted and meddlesome. That the test of the efficiency of a quarantine service should be its past record, and the confidence and approval of neighboring States and other quarantine officers. That the solution of the quarantine problem should be left to the local health authorities to be worked out in accordance with their individual requirements, and all progressive steps encouraged so long as such advances are made within the limits of safety. That the formulation of regulations by the United States Marine Hospital Service for control of State quarantine stations, without conference with the local quarantine officials, is to be deprecated, and can result only in conflict between State and National authorities. That the United States Marine Hospital Service has rendered valuable assistance in the way of collecting and disseminating information bearing on quarantinable disease, and that it can become otherwise useful by rendering assistance when called upon.

FINAL DISPOSAL OF GARBAGE AND REFUSE.

In connection with this matter, the following resolution was offered by Dr. M. S. Inglis, of Winnipeg:

"*Resolved*, That a committee of this Association be appointed by the Executive Committee for the purpose of promoting the collection and distribution of sanitary literature and health by-laws on, and regulations, to the members of this Association, to be known as the Committee on Distribution of Health Regulations and Reports. Said committee to have power to make such financial arrangements with members as will cover the cost of such distribution."

---

**Alcohol and Brain Work.**—It is a general impression that alcohol produces temporary ability for increased activity. Dr. Lauder Brunton asserts that "the influence of alcohol upon psychical processes is curious, for while it renders them much slower, the individual under its influence believes them to be much quicker than usual." The same fact is true of all stimulants. They give the individual the impression of greater vigor and strength, but this is simply a deception. Truly "wine is a mocker.'

